# Virus particle-based antibody-dependent cellular phagocytosis assay for HIV

**DOI:** 10.3389/fimmu.2026.1809342

**Published:** 2026-04-24

**Authors:** Chitra Upadhyay, Priyanka Gadam Rao

**Affiliations:** Division of Infectious Diseases, Department of Medicine, Icahn School of Medicine at Mount Sinai, New York, NY, United States

**Keywords:** ADCP, Fc-mediated activity, flow cytometry, HIV, THP-1 cells, virions

## Abstract

**Introduction:**

Antibodies mediate a wide range of antiviral functions, including neutralization and diverse Fc-dependent effector activities. Among these, antibody-dependent cellular phagocytosis (ADCP) has emerged as an important mechanism contributing to pathogen clearance, including during HIV-1 infection. Conventional bead-based ADCP assays typically rely on recombinant envelope glycoprotein (Env), which offers practical advantages but fails to fully recapitulate the native structural, conformational, and glycan features of virion-associated Env. This limitation reduces the physiological relevance of these assays for evaluating antibody function *in vivo*.

**Methods:**

We developed a virus particle-based ADCP assay designed to preserve the native membrane-embedded conformation and glycosylation of HIV-1 Env. The assay uses sucrose-purified, inactivated HIV-1 virions coupled to fluorescent beads as phagocytic targets, and the THP-1 human monocytic cell line as effector cells. The assay was optimized for sensitivity, reproducibility, and high‑throughput compatibility, and was applied to evaluate ADCP responses mediated by both monoclonal and polyclonal antibodies across multiple species.

**Results:**

The virus particle-based ADCP assay enabled robust and reproducible measurement of antibody-mediated phagocytosis in a biologically relevant antigen format. Using this system, we observed Env isolate-specific differences in Fc‑mediated activity that were not fully captured using recombinant gp120‑based assays. Direct comparison revealed that ADCP readouts obtained with recombinant Env proteins did not consistently mirror those measured using native virion-associated Env, highlighting qualitative differences in antibody engagement and Fc effector function depending on antigen presentation.

**Discussion:**

These findings demonstrate that preservation of native Env structure and glycosylation is critical for accurate assessment of Fc-dependent effector functions such as ADCP. The virus particle-based assay described here provides a practical and scalable framework for functional profiling of antibody responses in the context of HIV-1 infection and vaccination. By revealing antigen context-dependent differences in Fc‑mediated activity, this approach reinforces the importance of using antigenically authentic, virion-based systems to better reflect *in vivo* antibody function and inform vaccine and immunotherapeutic strategies.

## Introduction

Broadly neutralizing antibodies (bNAbs) are a major focus of HIV-1 vaccines and therapeutics due to their demonstrated ability to prevent infection and suppress viraemia in both preclinical and clinical settings. In nonhuman primate (NHP) and humanized mouse models, passive administration of bNAbs has been shown to provide protection against simian-human immunodeficiency virus (SHIV) and HIV-1 challenge respectively (1–7). Clinical trials, including the Antibody Mediated Prevention (AMP), have further validated this approach, demonstrating up to 75% protection against bNAb-sensitive HIV-1 strains in humans (8). Together, these findings highlight the central role of bNAbs as correlates of protection and highlight their potential as both preventive and therapeutic agents.

The predominant mechanism of bNAbs-mediated protection against HIV-1 is direct virus neutralization through Fab-mediated binding to the trimeric Env on intact virions, potentially blocking viral entry by preventing CD4 and coreceptor engagement or by disrupting the conformational changes required for membrane fusion (9–11). However, neutralization alone does not fully account for the protective activity observed across studies. Increasing evidence from both passive immunization and vaccine trials suggests that Fc-mediated effector functions such as antibody-dependent cellular phagocytosis (ADCP), antibody-dependent cellular cytotoxicity (ADCC), and complement activation, play an important and complementary role in antiviral efficacy by facilitating the clearance of infected cells and virions (1, 12–18). The RV144 trial, which showed modest efficacy, highlighted the potential importance of Fc−mediated functions in reducing HIV−1 acquisition, with FcγR−binding IgG responses and ADCC activity associated with decreased infection risk (19). Although ADCP was not assessed in the primary correlates analysis, subsequent studies demonstrated that vaccine−elicited antibodies were capable of mediating ADCP (20). The HVTN 505 trial extended these findings and identified ADCP as a correlate of reduced HIV acquisition (21, 22). Consistent with these data from human trials, NHP vaccine studies have similarly linked ADCP activity with reduced infection risk (23, 24). Together, these findings establish ADCP as an important Fc-mediated function associated with protective immunity.

The most widely adopted method to measure antibody mediated phagocytosis utilizes beads coated with HIV-1 Env proteins. The beads are opsonized by antibodies and subsequently internalized by monocytic cell lines such as THP-1 (25–30). This bead-based assay format is highly scalable and reproducible, enabling quantitative assessment of Fc-mediated phagocytic activity across diverse antibody specificities. However, because these assays rely on recombinant Env proteins (e.g., gp120, gp140, V3, or V1V2 proteins) (27–30), they oversimplify Env presentation and omit critical conformational and glycan features of the native trimer, limiting their ability to model virion−associated antigenic structure. An alternative strategy is to use infected cell-based assays, which provide greater physiological relevance by displaying native, membrane-embedded trimeric Env on the surface of infected target cells (31). Despite this advantage, cell-based assays are technically demanding, subject to variability in antigen density and cell viability (32–34) and less amenable to high-throughput implementation or cross-study standardization. Importantly, regardless of their distinct strengths and limitations, both bead−based and infected cell-based platforms share a major limitation: neither allows direct evaluation of Fc−mediated phagocytosis in the context of viral particle-associated Env (34, 35). In addition, although some studies have examined antibody−dependent phagocytosis of cell−free HIV−1, a comprehensive review highlights substantial variability across experimental systems and the absence of standardized methods for assessing HIV virion phagocytosis (36). Consistent with these challenges, Gach et al. reported minimal or no virion uptake across a wide range of monoclonal and polyclonal antibodies. The study attributed this to the inherently low density of Env protein on HIV−1 particles and the resulting insufficient FcγR cross−linking required for efficient phagocytosis (35). In contrast to these findings, Tay et al. showed that primary monocytes can internalize infectious HIV−1 in an antibody−dependent manner, with levels of uptake differing markedly by antibody isotype and subclass (26). However, their approach presents important limitations; the use of infectious, non−inactivated virions and reliance on spinoculation restricts both biosafety and scalability (26). In addition, the study relied on fluorescently tagged Gag, a modification that can interfere with viral assembly and Env incorporation due to the interaction between Gag and Env (37). Together, these factors highlight significant gaps in existing ADCP assay systems and underscore the need for platforms that maintain antigenic integrity without compromising feasibility or scalability.

Here, we report an ADCP assay that enables direct measurement of antibody-mediated phagocytosis of HIV-1 particles. By coupling native virions to fluorescent beads, this approach integrates the biological relevance of HIV-1 particles with the scalability and reproducibility of conventional bead-based platforms. It preserves native Env conformation and glycosylation while ensuring assay consistency and throughput. Together, these features make the virion−bead ADCP assay well−suited for vaccine evaluation, systems serology applications, and translational studies aimed at defining correlates of protective immunity. Overall, the platform provides a robust and adaptable tool for functional antibody profiling in both research and clinical settings.

## Materials and methods

### Cell lines

The HEK293T/17 (293T) were obtained from the American Type Culture Collection (ATCC, Manassas, VA). The following reagent was obtained through the NIH HIV Reagent Program, Division of AIDS, NIAID, NIH: TZM-bl Cells, ARP-8129, contributed by Dr. John C. Kappes, Dr. Xiaoyun Wu, and Tranzyme Inc (38). For all experiments, HEK293T/17 cells (293T) were used to produce infectious HIV-1 viruses and the TZM.bl cell line was used to assay virus infectivity. TZM.bl cell line is derived from HeLa cells and is genetically modified to express high levels of CD4, CCR5 and CXCR4 and contains reporter cassettes of luciferase and β-galactosidase that are each expressed from an HIV-1 LTR. The 293T and TZM.bl cell lines were routinely sub-cultured every 3 to 4 days by trypsinization and were maintained in Dulbecco’s Modified Eagle’s Medium (DMEM) supplemented with 10% heat-inactivated fetal bovine serum (FBS), HEPES pH 7.4 (10 mM), L-glutamine (2 mM), penicillin (100 U/ml), and streptomycin (100 μg/ml) at 37 °C in a humidified atmosphere with 5% CO2.

HEK293F cells (Thermo Fisher Scientific) were used to produce mouse mAbs in this study. The cells were cultured and maintained according to the manufacturer’s recommended protocols.

THP-1 cells are a human monocytic cell line commonly used in ADCP assays to model macrophage-mediated phagocytosis. THP-1 cells were purchased from ATCC and maintained in RPMI 1640 media (ATCC) containing 2 mM L-Glutamine (Gibco), 10% Fetal Bovine Serum (Sigma), 10 mM HEPES (Gibco), 55 μM beta-mercaptoethanol (Gibco), and 1X Penicillin/Streptomycin (Gibco). Cell culture densities were kept below 0.5 × 10^6^ cells/ml to maintain consistent assay performance.

### Plasmids

A full-length transmitted/founder (T/F) infectious molecular clone (IMC) of pREJO.c/2864 (REJO, ARP-11746) was obtained through the NIH HIV Reagent Program, Division of AIDS, NIAID, NIH, contributed by Dr. John Kappes and Dr. Christina Ochsenbauer (39). REJO is a tier 2, clade B, T/F isolate. The IMC of RHPA and QH0692 (tier 2, clade B, T/F) were kindly provided by Dr Benjamin Chen. The IMCs were generated by cloning the Env into a pNL4.3 backbone to construct pNL-RHPA and pNL-QH0692, respectively. The CMU06 IMC was generated similarly by cloning the Env into a pNL4.3 backbone to construct pNL-CMU06 (40).

### Antibodies and plasma samples

The following antibody reagents used in this study were obtained through the NIH AIDS Reagent Program, Division of AIDS, NIAID, NIH: Anti-HIV-1 gp120 monoclonal VRC01 from Dr. John Mascola (41); anti-HIV-1 gp120 monoclonal antibody NIH45–46 G54W, contributed by Dr. Pamela Bjorkman (42); anti-HIV-1 gp41 monoclonal 2F5 from Polymun Scientific (43); polyclonal HIV-Ig contributed by Dr. Luiz Barbosa. The V2i (830A) and V3 (2219) and the irrelevant (non-HIV-1) anti-anthrax 3685 mAbs were obtained from the laboratory of Dr. Susan Zolla-Pazner (44–52). The mAb 3685 was used as a negative control. HIV-1 positive human plasma samples used were a gift from Dr Colleen Courtney (53). The mouse serum samples were from animals co-immunized with gp120 DNA and protein immunogens (30). Mouse monoclonal antibodies were generated by hybridoma technology, where splenic B cells of mouse immunized with gp120 immunogens were fused with myeloma cells to create antibody-secreting hybridomas. For production and purification, the antibody sequences (heavy and light chains) were cloned into the mammalian expression vector pcDNA3.1. These plasmids were then co-transfected into HEK293F cells for protein expression (data not shown). The transfected cells expressed the recombinant antibodies, which were subsequently harvested from the culture supernatant, purified and used as reagents in this study.

### Virus production and purification

Infectious viruses were generated by transfecting 293T cells with pREJO, pNL-CMU06, pNL-RHPA and pNL-QH0692 plasmids using jetPEI transfection reagent (Polyplus, New York, NY) (54). Supernatants were harvested after 48 hours and clarified by centrifugation and 0.22μm filtration. Virus infectivity was assessed on TZM.bl cells as described (40, 54). Briefly, serial two-fold dilutions of virus stock in 10% DMEM were incubated with TZM.bl cells (in duplicates for each dilution) in half-area 96-well plates in the presence of DEAE-dextran (12.5 μg/ml) for 48 hours at 37 °C. Virus infectivity was measured by β-galactosidase activity (Promega, Madison, WI). Virus stocks were concentrated (20X) by ultracentrifugation over 20% (w/v) sucrose in 1X phosphate buffered saline (PBS) at 25,000 RPM for 2 hours in an SW-28 swinging bucket rotor (Sorvall, Thermofisher Scientific). Supernatants were decanted and pellets dried briefly before resuspension in PBS. Inactivation of virions was carried out using Aldrithiol-2 (AT-2) (55, 56). Briefly, 125 ul of sucrose-purified virus was incubated with 0.5 mM AT-2 in DMSO for 2 hours at 37 °C, followed by centrifugation at 13,000 rpm for 2 hours. The supernatant was discarded, and the pellet re-suspended in 125 ul PBS. Inactivation was confirmed by measuring infectivity in TZM.bl cells and Env content was checked by Western blotting.

### Western blotting

To quantify and monitor the expression of Env in each virus preparation Western blot analyses were performed as described before (57). Briefly, the sucrose-purified virus particles were lysed, resolved by SDS-PAGE on 4-20% tris-glycine gels (Bio-Rad, Hercules, CA), and blotted onto membranes, which were then probed with antibodies. A cocktail of anti-human anti-gp120 MAbs (anti-V3: 391, 694, 2219, 2558; anti-C2: 841, 1006; anti-C5: 450, 670, 722; 1μg/ml each) was used to detect Env. MAb 91-5D (1μg/ml) was used to detect Gag p24. Membranes were developed with Clarity Western ECL Substrate (Bio-Rad, Hercules, CA) and imaged by iBright FL1500 Imaging System (Invitrogen, Carlsbad, CA). Purified recombinant gp120 and p24 proteins were also loaded at a known concentration as controls and quantification standards. Band intensities were quantified using the iBright Analysis Software Version 5.0.1 (Invitrogen, Carlsbad, CA).

### Coupling of fluorescent beads to virions

The coupling strategy used in this study is a standard method widely used in Luminex based multiplex immunoassays and is published previously (19, 58–60). Here we applied this approach for coupling the virus to the beads (57). Sucrose-purified, inactivated virions were covalently coupled to carboxylate-modified FluoSphere microspheres (1.0 µm) using a two-step carbodiimide reaction with the xMAP Ab Coupling (AbC) Kit according to manufacturers’ instructions (Luminex, Austin, TX). Carboxylated beads purchased from Thermo Fisher (cat# F8823; 505/515 nm), were coupled to 125 μl of 20X concentrated virus preparations. Briefly, the stock microspheres were vortexed and sonicated to resuspend and disperse the microspheres and 12 μl (~36.4x10^9^ beads) was transferred to a tube containing 1200 ul of 1% BSA/1X PBS (per virus). The microspheres were washed twice with 500 μl of activation buffer followed by vortexing and sonication after each step. The microspheres were activated with 400 μL of activation buffer, 50 μL of 50 mg/ml Sulfo-NHS (N-hydroxysulfosuccinimide), 50 μL of 40 mg/mL ethyl dimethylaminopropyl carbodiimide hydrochloride (EDC) and incubated for 20 min at room temperature with end-to-end rotation. Carbodiimide crosslinking (EDC/Sulfo−NHS chemistry) activates carboxyl groups on fluorescent beads, converting them into amine−reactive NHS esters. When HIV particles are added, primary amines on virion surface proteins or host−derived membrane proteins, incorporated into the virion membrane, react with these activated esters to form stable covalent amide bonds. This zero−length crosslinking method efficiently couples intact HIV virions to the bead surface without requiring additional linker molecules. The microspheres were washed three times in activation buffer, then incubated with AT-2 inactivated virus in activation buffer for 2 hours at room temperature. We typically used a volume of 20X concentrated virus that equals ~175ng total as measured by Western blotting. The microspheres were subsequently washed and resuspended in 1.2 mL of 0.1% BSA/PBS and stored at 4 °C until ready to use. This volume is further diluted 1:1 and is sufficient to run the assay on two 96−well plates.

Based on the theoretically estimated Env content of HIV-1 virions (~10–14 Env trimers per particle, corresponding to ~30–40 gp120 molecules per virion), coupling 175 ng of virion-associated Env to approximately 3.6 × 10^10^ beads corresponds to a theoretical maximum of ~2-3×10^10^ virions per reaction, or on the order of one virion per bead. Because this estimate assumes complete recovery and 100% coupling efficiency, the actual number of virions per bead is likely lower. Based on these theoretical considerations, the bead-to-virus ratio was selected to minimize the likelihood of multiple virions conjugating to a single bead and to favor low-density Env presentation that more closely approximates native viral conditions.

### ADCP assay protocol

The coupled microspheres were diluted 1:1, dispensed at 10 µl/well and incubated with serial dilutions of monoclonal antibodies or plasma (10 µl/well) for 2 hours at 37 °C. Antibody opsonized virions coupled microspheres were washed with 0.1% BSA/PBS twice to remove unbound antibodies. THP-1 cells were added (200 μL/well) at a concentration of 1.25 × 10^5^ cells/mL (2.5 × 10^4^ cells/well) and incubated with the immune complexed microspheres overnight for ~16 h at 37 °C. Cells were then fixed with 4% PFA and acquired on a Attune NxT flow cytometer.

Data analysis was performed using FCS Express 7 Research Edition (*De Novo* Software) as follows: Cells were gated on a plot of forward scatter area versus side scatter area (FSC-A vs SSC-A). Doublets were excluded using a forward scatter height versus area plot (FSC-H vs FSC-A). Geometric mean fluorescence intensity (MFI) values of AF488 cells, representing phagocytosed virus particles coupled to yellow green microspheres, were determined. ADCP scores were calculated as follows: [(% microsphere positive cells) x (MFI of the microsphere positive cells)/10,000]. Control wells included virion coupled beads alone, cells alone and virion coupled beads without Ab and cells (no Ab control for background phagocytosis).

### Statistical analysis

Statistical analyses were performed as indicated in the figure legends with one or two-way ANOVA using Dunnett multiple comparison test using GraphPad Prism 10.6.0. Statistical significance was interpreted in conjunction with effect size and dose-response behavior to avoid overinterpretation of small but consistent differences.

Intra-assay precision was calculated as the coefficient of variation (CV%) between technical replicates within a single experiment, and inter-assay reproducibility was calculated as the CV across independent experiments performed on different dates using the mean replicate value per run.

## Results

### Development of a virus particle-based ADCP assay

**Figure 1:**
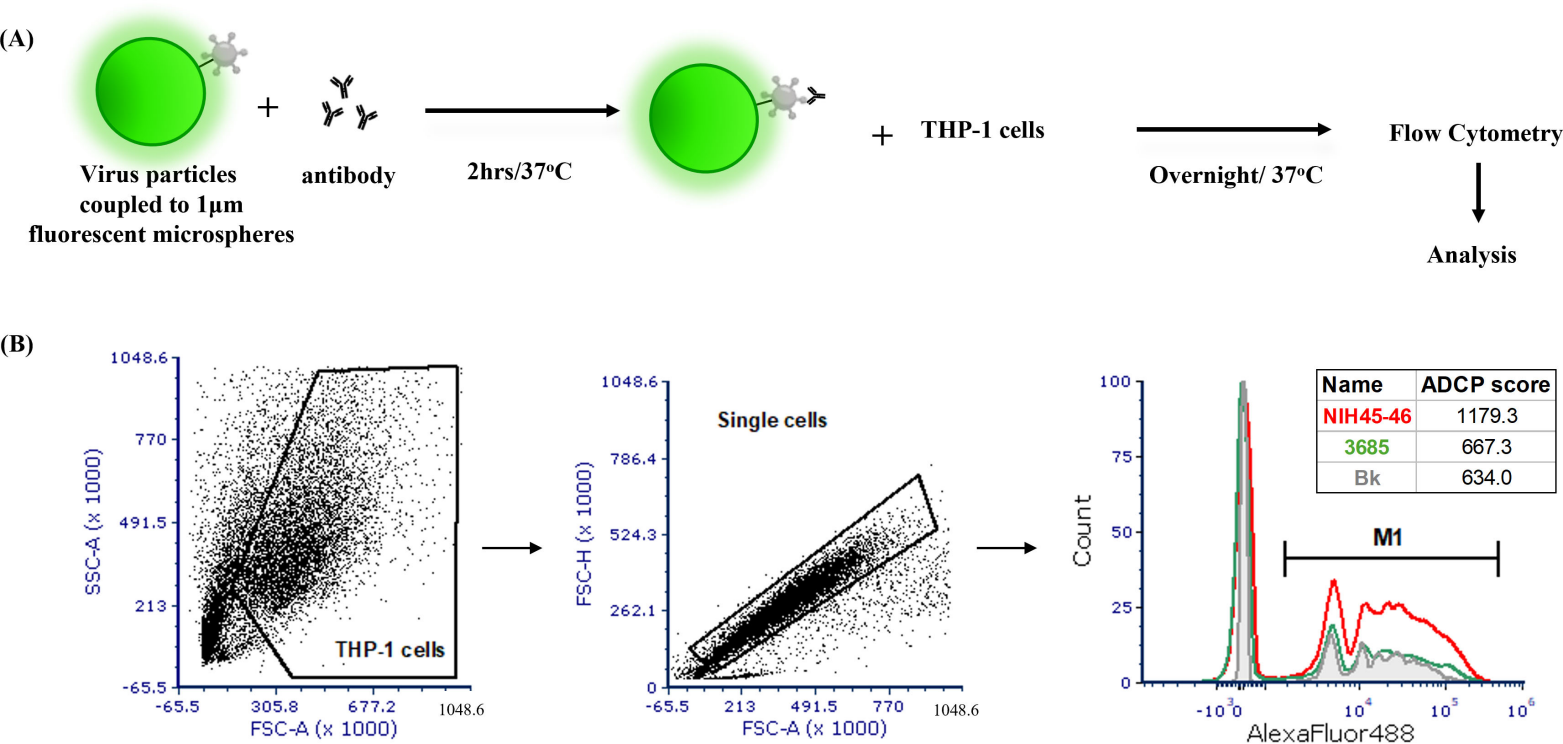
ADCP assay for detecting phagocytosis of antibody-opsonized HIV-1 virions. **(A)** Schematic overview of the assay. The schematic is made to depict a 1:1 bead-virion input ratio used during the coupling reaction; the illustration is conceptual and not intended to represent actual virion stoichiometry per bead. Sucrose pelleted RHPA virions were carboxyl-coupled to 1 μm yellow-green beads. Virion-coupled bead were then incubated with HIV-1 monoclonal antibodies (mAbs), washed, incubated with human THP-1 monocytes, fixed, and analyzed by flow cytometry. **(B)** Gates were drawn on singlet THP-1 cells, and phagocytic scores were calculated from data on the AlexaFluor488 fluorescence channel to quantify envelope-specific ADCP. Histograms indicate results with 5 μg/ml positive (NIH45-46; red) and negative control (3685-non-HIV-1; green) mAbs. Background phagocytosis (Bk) of virion-couple beads with no Ab is shown in gray.

To enable biologically relevant measurement of Fc effector activity against HIV-1, we developed a virus particle-based ADCP assay. As shown in **Figure 1A**, sucrose-pelleted RHPA virions were coupled to 1 μm yellow-green, fluorescent beads via a two-step carboxyl chemistry. This coupling strategy produced phagocytosis−competent targets while maintaining the native, membrane−associated presentation of Env on intact virions. The resulting virion−coated beads were opsonized with antibody and incubated with THP-1 monocytic effector cells. After fixation, phagocytosis was quantified by flow cytometry using AF488 fluorescence to detect bead uptake by THP-1 cells. Gating was performed on singlet THP-1 cells to exclude aggregates, and ADCP scores were calculated by multiplying the geometric mean fluorescence intensity (MFI) of bead-positive cells by the percentage of bead-positive cells, normalized to background.

In our previously published study (57), we optimized the amount of Env used for bead coupling to balance sensitivity with a low signal-to-noise ratio, ensuring clear discrimination between positive and negative antibody binding. The present ADCP assay used this same optimized Env input. For initial studies, we used RHPA, a T/F, clade B, tier 2, HIV-1 isolate and monoclonal antibodies (mAb) targeting the conformational CD4bs (NIH45-46) epitope on Env. A non-HIV-1 mAb 3685 was used as a negative control. As shown in **Figure 1B**, the mAb NIH45-46 (red histogram) mediated robust phagocytic activity with an ADCP score of 1179.3, which was higher than both the non-HIV-1 control mAb 3685 (green histogram; ADCP score = 667.3) and the background control with no antibody (gray histogram; ADCP score = 634.0). These data demonstrate that the assay reliably detects Fc-mediated phagocytosis of HIV-1 antibody−opsonized virions with high specificity and sensitivity.

### Detection of ADCP activity by human monoclonal antibodies

**Figure 2:**
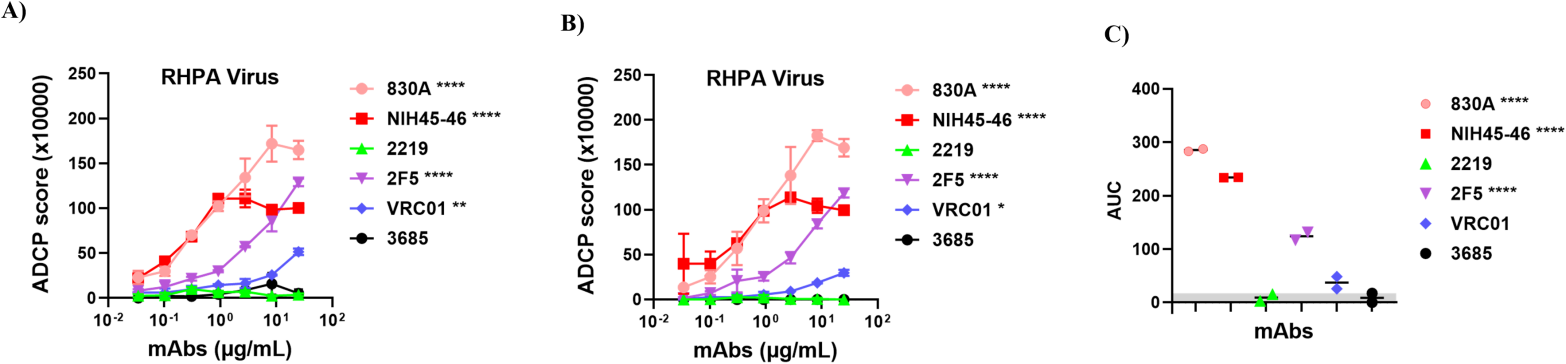
ADCP assay for detection of phagocytosis of human antibody opsonized virions. Sucrose pelleted RHPA virions were carboxyl-coupled to 1 μm yellow-green beads. Virion-coupled bead were then incubated with HIV-1 envelope-specific mAbs, washed, incubated with human THP-1 monocytes, fixed, and analyzed by flow cytometry. Beads incubated with cells without Ab were used as background and subtracted. An irrelevant non-HIV-1 mAb specific to anthrax (3685) was used as negative control. **(A)** Experiment 1 and **(B)** Experiment 2 showing dose-response curves of ADCP activity of HIV-1 mAbs against RHPA virions across a 3-fold dilution series starting at 25 μg/mL. Data are shown as mean ± SD from replicate wells. **(C)** Area under titration curve (AUC) values calculated from **(A, B)** Gray shading indicates the AUC for the negative control mAb (3685). Each dot represents an independent experiment, and the horizontal line indicates the median. For **(A, B)**, statistical significance was determined using a two-way ANOVA with Dunnett’s multiple-comparisons test, comparing each mAb to the negative control (3685) based on the main column effect averaged across all concentrations. Significance levels are indicated as *p<0.05; **p<0.01; ****p<0.0001. Values with p > 0.05 are unmarked. For **(C)**, a one-way ANOVA with Dunnett’s multiple-comparisons test was used to compare the AUC of each mAb to the negative control (3685).

We next applied the assay to evaluate the functional capacity of a panel of well-characterized human HIV-1 mAbs targeting diverse HIV-1 Env epitopes including the CD4 binding site (VRC01, NIH45-46), gp41 (2F5), V2 (830A), and V3 linear epitopes (2219). Each mAb was tested across a range of concentrations using RHPA virus particles coupled to fluorescent beads and THP-1 monocytes. As shown in **Figures 2A, B**, several HIV-1-specific mAbs mediated dose-dependent phagocytosis. Among these, NIH45–46 and 830A demonstrated the strongest ADCP activity, followed by moderate responses from VRC01 and 2F5. As expected, the irrelevant anthrax-specific mAb 3685 showed no detectable activity, confirming assay specificity. The V3 mAb 2219 did not mediate detectable ADCP. Quantitative analysis of the area under the titration curves (AUC) from both experiments (**Figure 2C**) confirmed clear differences in ADCP potency across the panel, with NIH45–46 and 830A yielding the highest values. The irrelevant anthrax-specific mAb 3685 remained at baseline throughout, validating assay specificity.

### Broad applicability across viral strains and antigens

**Figure 3:**
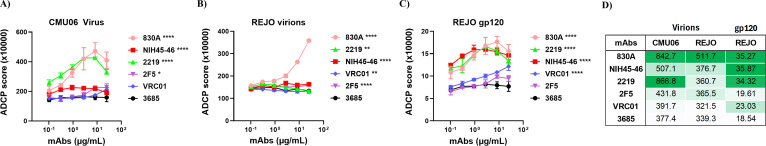
Broad applicability of the ADCP assay across HIV-1 strains and antigen formats. Sucrose pelleted virions were carboxyl-coupled to 1 μm yellow-green beads. Virion-coupled bead were then incubated with HIV-1 envelope-specific mAbs, washed, incubated with human THP-1 monocytes, fixed, and analyzed by flow cytometry. ADCP with **(A)** CMU06 virions, **(B)** REJO virions and **(C)** Rejo gp120. Beads incubated with cells without Ab were used as background and subtracted. An irrelevant non-HIV-1 mAb specific to anthrax (3685) was used as negative control. Data represent the mean ± SD of replicate measurements from one experiment. **(D)** Area under the curve (AUC) values were calculated from **(A-C)** Color-code is relative to the AUC of the negative control mAb (3685) for each antigen format, with darker green indicating larger effect sizes compared to 3685. Higher AUC values reflect stronger phagocytic activity. Statistical significance was determined using two-way ANOVA with Dunnett’s multiple-comparisons test comparing each mAb to the negative control (3685) based on the main column effect averaged across concentrations (*p < 0.05; **p < 0.01; ****p < 0.0001). Values with p > 0.05 are unmarked.

Next, we tested the same mAb panel against virions from two additional HIV-1 isolates, CMU06 and REJO, as well as recombinant REJO gp120 protein. With CMU06 virions (**Figure 3A**), mAbs 830A and 2219 mediated robust, dose-dependent ADCP. NIH45–46 and 2F5 displayed moderate activity above background, whereas VRC01 remained near baseline, similar to the irrelevant control mAb 3685. When tested against REJO virions (**Figure 3B**), mAb 830A retained strong dose-dependent phagocytic activity, while NIH45–46 and 2F5 showed only low activity. V3 mAb 2219, which was effective against CMU06, exhibited minimal activity. These findings from **Figures 2**, **3** highlight strain-dependent differences in antibody-mediated phagocytosis, consistent with variation in Env epitope accessibility between viral isolates (57) and underscore the importance of testing multiple HIV-1 strains rather than relying on a single viral isolate.

We next assessed ADCP activity against recombinant Env by testing the panel with REJO gp120 monomers coupled to beads (**Figure 3C**). In this format, mAbs 830A, NIH45-46, and 2219 mediated the strongest phagocytic responses. VRC01 again showed weak activity and as expected, the gp41-specific mAb 2F5 showed no activity. Because antigen density and presentation differ substantially between virion and protein coupled beads, direct quantitative comparison of ADCP magnitude across antigen formats were not performed. Instead, we focused on qualitative differences in antibody response patterns. As anticipated, the gp120-based assay did not replicate the restricted ADCP pattern seen with REJO virions. Antibodies that were weak against REJO virions, such as 2219 and NIH45-46, mediated strong phagocytosis with gp120-coupled beads. This divergence reflects the inherent structural differences between native, membrane−embedded Env trimers on virions and soluble monomeric gp120, including altered quaternary structure and increased exposure of normally occluded regions, such as V3.

To summarize differences in ADCP magnitude within each antigen format, we calculated the area under the curve (AUC) for each mAb. **Figure 3D** presents a color-coded comparison of AUC values relative to the mAb 3685, providing a visual summary of phagocytic activity across antigen contexts. For CMU06 virions, 2219 and 830A showed the highest AUC values, followed by NIH45−46, 2F5, VRC01, and 3685. For REJO virions, 830A exhibited the highest AUC, while NIH45−46, 2F5, 2219, VRC01, and 3685 formed a lower−activity group. For REJO gp120, NIH45−46, 830A, and 2219 displayed the highest AUC values, whereas VRC01 and 2F5 showed lower activity near background levels defined by 3685.

Using two-way ANOVA with Dunnett’s multiple-comparisons test, all HIV-1 specific mAbs showed statistically significant increases in mean ADCP activity relative to the negative control (3685) when averaged across concentrations. However, statistical significance did not always correspond to large biological effects. Notably, the magnitude of enhancement differed substantially among antibodies, with mAb 830A exhibiting the strongest and most consistent activity, whereas other antibodies showed smaller effect sizes despite statistical significance. These results emphasize that statistical significance alone may not fully capture biologically meaningful differences in Fc-mediated effector function, particularly when antigen presentation varies across formats.

### Polyclonal HIV-1 infected sera mediate virion phagocytosis

**Figure 4:**
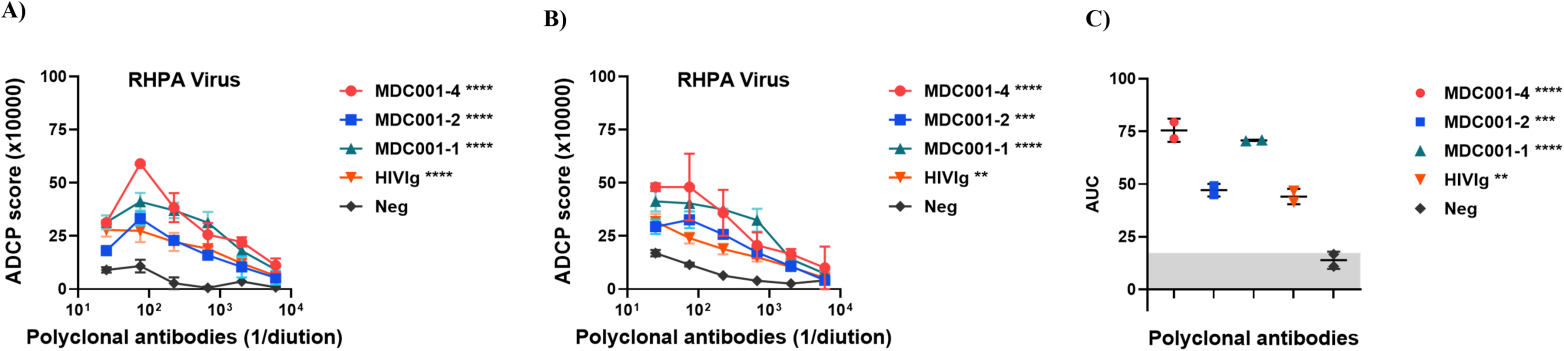
ADCP assay to measure phagocytosis of HIV-1 virions opsonized with polyclonal antibodies from infected individuals. Sucrose pelleted RHPA virions were carboxyl-coupled to 1 μm yellow-green beads. The virion-coupled bead were incubated with heat-inactivated sera from HIV-1 infected individuals and polyclonal IgG (HIVIg), washed, then incubated with human THP-1 monocytes. After fixation samples were analyzed by flow cytometry. Virion coupled beads incubated with cells in the absence of Ab served as background and were subtracted. Sera from healthy individual (Neg) was used as negative control. **(A)** Experiment 1 and **(B)** Experiment 2. **(C)** Area under titration curve (AUC) values were calculated from **(A, B)** Gray shading indicates the AUC range for the negative control polyclonal antibody (Neg). For **(A, B)**, statistical significance was assessed using two-way ANOVA with Dunnett’s multiple-comparisons test, comparing each mAb to the negative control (3685) based on the main column effect averaged across concentrations. Significance is indicated **p<0.01; ***p<0.001; ****p<0.0001. Values with p > 0.05 are unmarked. For **(C)**, one-way ANOVA with Dunnett’s multiple-comparisons test was used to compare AUC values for each antibody relative to the negative control (3685). Each dot represents an independent experiment, and horizontal lines denote median values.

We next assessed whether the assay developed could be used to detect ADCP mediated by polyclonal antibodies. RHPA virion-coupled fluorescent beads were incubated with heat-inactivated sera from HIV-1 infected individuals, as well as with purified polyclonal immunoglobulin G (HIVIg) followed by incubating with THP-1 monocytes as phagocytic effector cells. As shown in **Figures 4A, B**, sera from infected donors consistently mediated dose-dependent ADCP activity, whereas HIV-1 negative sera (Neg) showed minimal activity. Quantitative analysis of AUC confirmed significant higher ADCP activity in HIV-1 infected sera compared to negative control (**Figure 4C**). These findings demonstrate that naturally elicited polyclonal antibodies in chronic HIV-1 infection can drive Fc-mediated clearance of virions and validate the sensitivity and biological relevance of the virion-based ADCP assay for profiling humoral immunity.

### Extension of the assay to mouse monoclonal antibodies and sera

**Figure 5:**
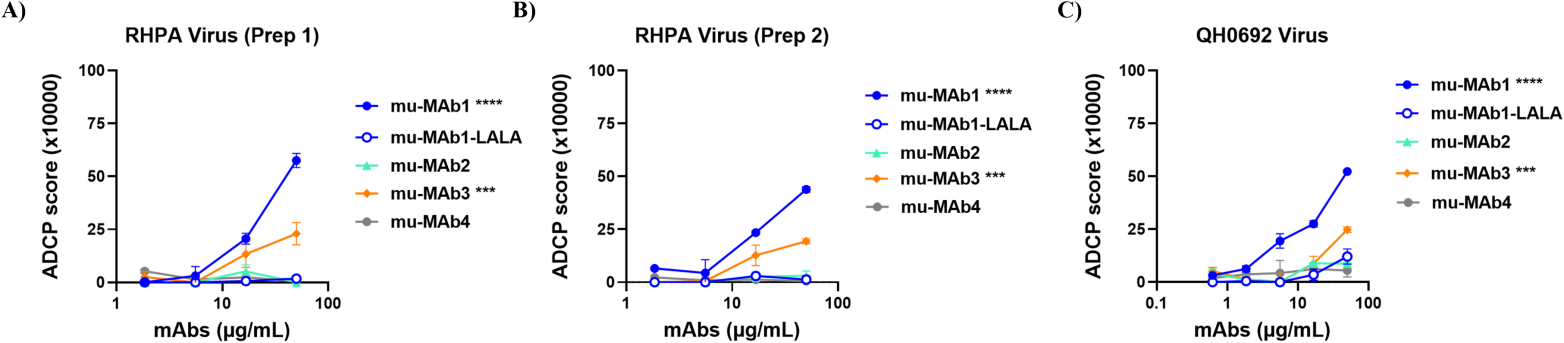
ADCP activity of murine monoclonal antibodies against HIV-1 virion-coupled beads. Sucrose pelleted RHPA virions, coupled to 1 μm yellow-green beads were incubated with HIV-1 envelope-specific mouse mAbs, washed, incubated with human THP-1 monocytes, fixed, and analyzed by flow cytometry. An Fc-impaired variant of mu-MAb1 (mu-MAb1-LALA) and an irrelevant murine monoclonal antibody (mu-MAb4) were included as negative controls. RHPA viruses from two different transfections are tested **(A)** Prep 1 **(B)** Prep 2). **(C)** Another T/F isolate QH0692 was tested similarly. Beads incubated with cells without Ab were used as background and subtracted. Data represent the mean ± SD of replicate measurements from one experiment. Statistical significance was determined using a two-way ANOVA with Dunnett’s multiple-comparisons test, comparing each mAb to the negative control (mu-MAb4) based on the main column effect averaged across all concentrations. Significance levels are indicated as ***p<0.001; ****p<0.0001. Values with p > 0.05 are unmarked.

To expand the applicability of the ADCP assay to preclinical models, we tested murine (mu) Env-specific mAbs against RHPA and QH0692 virion-coupled beads. RHPA virions from two independent preparations (Prep 1 and Prep 2) and QH0692 virions were coupled to fluorescent beads, opsonized with mouse mAbs, and incubated with THP-1 monocytes. As shown in **Figures 5A, B**, mu-MAb1 mediated robust dose-dependent ADCP activity against RHPA virions, with consistent results across both virus preparations. The mu-MAb3 also mediated detectable ADCP, although with lower magnitude than mu-MAb1 whereas, mu-MAb2 did not show any activity. The irrelevant control antibody mu-MAb4 similarly lacked activity. Specificity was further supported by the Fc-impaired mu-MAb1 LALA variant, which failed to mediate ADCP (61–63). The assay also performed well with QH0692 virions (**Figure 5C**), demonstrating phagocytic responses for Env−specific mu−MAb1 and mu−MAb3 antibodies, while mu−MAb1−LALA and mu−MAb4 remained at background.

Notably, mu-MAb1, mu-MAb2, and mu-MAb4 are of the IgG2a isotype, whereas mu-MAb3 is IgG1. Because murine IgG2a antibodies bind activating Fcγ receptors with higher affinity than IgG1, these results are consistent with known isotype-dependent differences in Fc-mediated effector function (64). Together, these data demonstrate that the virion-based ADCP assay is compatible with murine antibodies, reproducible across independent virus preparations, and applicable to distinct HIV-1 isolates, supporting its use for functional antibody assessment in animal models.

**Figure 6:**
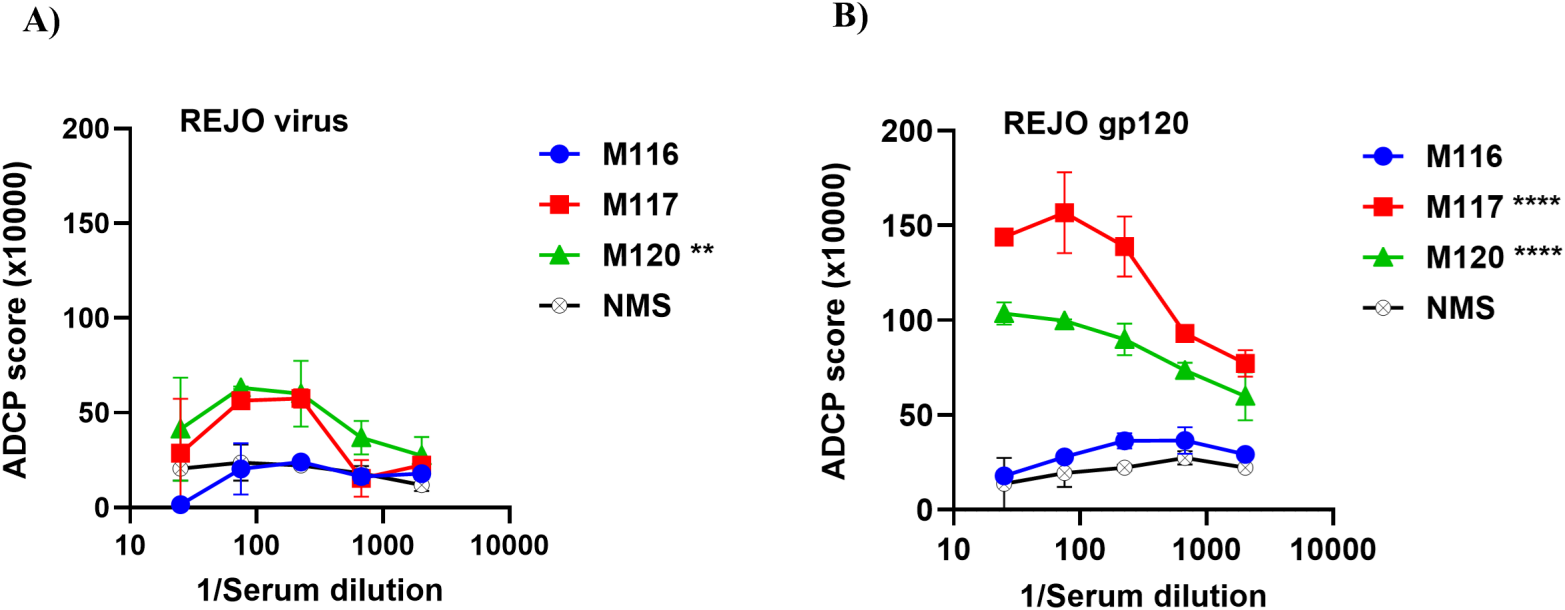
Antibody-dependent cellular phagocytosis mediated by sera from immunized mice against REJO HIV-1 virions and gp120. Sera from mice co-immunized with HIV-1 gp120 DNA + protein were tested for their ability to mediate ADCP using phagocytic THP-1 cells and fluorescent beads coated coupled to either AT-2 inactivated **(A)** REJO virions or **(B)** REJO gp120. ADCP scores are shown for individual vaccinated animals (M116, M117, M120) and normal mouse serum (NMS) as a negative control. Data represent the mean ± SD of replicate wells from one experiment. Statistical significance was determined using two-way ANOVA with Dunnett’s multiple-comparisons test comparing each vaccinated serum to NMS based on the main column effect averaged across dilutions **P < 0.01; ****P < 0.0001). Values with p > 0.05 are unmarked.

To assess the performance of the assay in a vaccination setting, we measured ADCP activity in serially diluted sera from mice co−immunized with HIV−1 gp120 DNA and protein (30). As shown in **Figure 6A**, vaccinated animals displayed detectable phagocytic responses against REJO virions, with individual mice (M116, M117, M120) exhibiting distinct magnitudes compared with normal mouse serum (NMS). A similar pattern was observed when ADCP activity was assessed using gp120−coupled beads (**Figure 6B**), where M117 and M120 showed particularly strong and statistically significant responses across multiple dilutions. Together, these results demonstrate that the virion−coupled ADCP assay reliably detects vaccine−elicited functional antibodies and complements gp120 protein-based ADCP measurements in preclinical immunogenicity studies.

### Assay precision and reproducibility

To evaluate the robustness of the virion-bead ADCP assay, we assessed both intra-assay precision and inter-assay reproducibility across three independent experiments using the mouse mAbs shown in **Figure 5**, each tested with RHPA virions derived from independent virus preparations (**Supplementary Table 1**). Each antibody-virion condition was tested in duplicate wells in every run.

Intra-assay precision: Across the three experiments, conditions yielding measurable phagocytic activity showed low to moderate variability, consistent with expectations for flow-based cell assays. For antibodies with quantifiable signals, intra-assay coefficients of variation (CV%) generally ranged from 2.8% to 10.6%, as seen with mu-MAb1, mu-MAb2 (CV 3.3-73.7%, signal-dependent), and mu-MAb3 (CV 6.2-141.45%). As expected, CVs increased substantially when mean activity approached background levels, including the Fc-impaired mu-MAb1-LALA variant and the negative control antibody. Overall, these data demonstrate strong within-run repeatability for biologically meaningful signal ranges.

Inter-assay reproducibility: To assess run-to-run consistency, mean ADCP scores for each antibody were compared across the three independent experiments. Inter-assay CVs ranged from 45.7% to 79.0% for antibodies exhibiting measurable activity, reflecting expected day-to-day variability associated with independent virus preparations and assay conditions. Importantly, despite variation in absolute signal magnitude, the relative ranking of antibody activities was highly preserved across experiments, indicating reproducible discrimination of functional differences among antibodies.

Together, these analyses demonstrate that the virion-bead ADCP assay exhibits strong intra-assay precision and reliable inter-assay reproducibility for relative potency ranking, across independent experiments and virion preparations. This performance profile is consistent with other complex, cell-based Fc-effector assays, where absolute signal intensity may vary between experiments, but functional ordering of antibodies is maintained.

## Discussion

In this study, we describe a virion-coupled ADCP assay that complements existing ADCP approaches by using native, membrane-embedded Env as the phagocytic target. By evaluating virions from multiple HIV-1 isolates and antibody samples encompassing both monoclonal and polyclonal responses from vaccination and infection settings, we show that the assay reproducibly measures Env-directed phagocytic activity. Comparisons between virion-based and gp120-coated bead formats revealed differences in ADCP profiles for select antibodies, indicating that virion-associated Env can capture functional features not reflected in soluble protein-based assays. Because Env is maintained in its trimeric, glycosylated form within a membrane context, the virion-coupled assay provides a biologically relevant platform for ADCP assessments. Together, these findings support the inclusion of virion-based readouts into preclinical immunogenicity studies and highlight the importance of antigen presentation on Fc-mediated effector function.

Fc-mediated effector functions of antibodies, including ADCP, are increasingly recognized as important contributors to protective immunity against HIV-1, complementing Fab-mediated virus neutralization, with multiple nonhuman primate studies linking higher ADCP activity to reduced infection risk (23, 24). While ADCP was not included in the primary correlates analysis in RV144, the only trial that showed modest efficacy, follow-up studies demonstrated that vaccine-elicited antibodies were capable of mediating ADCP (20), and analyses from HVTN 505 further supported ADCP as an immune correlate associated with reduced HIV acquisition (21, 22). Most ADCP studies rely on bead-based assays coated with recombinant Env proteins as phagocytic targets. These assays offer scalability and reproducibility, making them well suited for large comparative studies, however, they do not fully capture the native virion context, particularly membrane anchoring, structural organization and the heterogeneous glycan complexity of the native Env trimer on virions, features that can influence antibody binding and Fc-mediated effector function *in vivo* (25, 26, 34). Infected cell-based assays can present Env in a native physiologically relevant membrane setting, but they are technically demanding and often variable in antigen density and composition, complicating standardization and broader application (31). Together, these limitations highlight the need for assay platforms that incorporate authentic virion-associated Env as phagocyte target.

An important intermediate approach has been the use of native like soluble trimers (e.g., SOSIP) in Fc effector assays, since these antigens preserve many features of the prefusion Env spike and have enabled robust detection of ADCP in both preclinical and clinical contexts. For example, Martin et al. used biotinylated BG505 SOSIP trimers immobilized on beads and THP-1 effector cells to demonstrate robust ADCP by rhesus macaque mAb (65). In clinical vaccine studies, serum antibodies elicited by ConM SOSIP.v7 mediated ADCP of SOSIP-conjugated beads (66), and polyclonal IgG from HIV-infected individuals showed binding to trimeric SOSIPs that correlated with phagocytic activity (67). However, despite these strengths, SOSIP trimers remain imperfect surrogates for virion Env. They lack the native membrane context, do not include the full gp41 ectodomain/MPER and transmembrane regions, and the stabilizing mutations (e.g., SOS and I559P) may shift conformational dynamics away from the pretriggered State 1 ensemble (68). Moreover, SOSIPs display less complex glycans than those on virion Env, likely related to both intrinsic stabilization and the absence of membrane tethering (69, 70). Because epitope exposure and glycan composition can alter binding by both neutralizing and non-neutralizing antibodies, and potentially downstream Fcγ receptor engagement, these differences are particularly relevant when interpreting Fc effector readouts. Additionally, the membrane itself influences Env conformation, with detectable differences in gp120 and gp41 regions between membrane−bound and SOSIP forms (71). Finally, not all HIV-1 isolates can be readily engineered or stably produced as SOSIP trimers, restricting their use to a limited subset of strains, an important consideration given the strain specific differences in Fc effector activity that we observed with virion targets.

Prior work using infected cell systems has provided key insights into Fc mediated clearance mechanisms, including the observations that bNAbs can drive efficient ADCP of infected cells whereas many non-neutralizing antibodies do not (34). However, infected cell assays do not directly interrogate antibody mediated uptake of cell free virions and may be influenced by the presence of uncleaved, nonfunctional Env forms on the cell surface that are largely excluded from virions (72), potentially complicating direct extrapolation to virion clearance. Tay et al. examined antibody dependent internalization of infectious virions and highlighted the roles for antibody isotype/subclass in shaping uptake (26). While informative, this approach used infectious virus, that are not inactivated, and relied on spinoculation, introducing technical constraints and limiting scalability. In this context, the assay described here provide a practical way to interrogate Fc dependent phagocytosis against authentic virion associated Env while maintaining safety and experimental tractability for comparative studies.

The virion−based ADCP assay described here integrates native Env presentation with the practicality of a bead−based system. In this format, inactivated virions are covalently attached to fluorescent beads and used as phagocytic targets. Although beads are substantially larger than HIV−1 particles, the goal is not to model virion size but to retain the native Env architecture that governs antibody binding and Fcγ receptor engagement. This strategy aligns with prior work demonstrating that both antigen organization and Fc-Fcγ receptor interactions strongly influence phagocytic outcomes (26). By displaying intact virions on a uniform scaffold, the assay offers a reproducible, scalable readout of antibody−mediated phagocytosis without the variability and biosafety challenges associated with free or infectious virus.

Our findings differ from those of Gach et al., who reported that HIV−1 virions are largely resistant to ADCP due to low Env trimer density on the virion surface (35). In our hands, and in agreement with observations by others (26), virions were readily phagocytosed in the presence of diverse monoclonal and polyclonal antibodies. The virion input used for coupling (~175 ng Env per reaction) corresponds to a theoretical average of approximately one virion per bead, making it unlikely that the bead format artificially increases functional Env density (see Methods). Instead, we suggest that coupling of virions to beads improves the uniform accessibility of existing Env trimers, facilitating productive Fcγ receptor engagement without modifying intrinsic virion properties. This is supported by the behavior of the V3−specific mAb 2219, which exhibited no ADCP activity against REJO and RHPA virions, consistent with the native occlusion of V3 in the closed Env trimer (57, 73–75). In contrast, 2219 mediated robust ADCP when targeting REJO gp120, where the V3 loop is fully exposed. The mAb 2219 also exhibited measurable activity with CMU06 virions, indicating that epitope accessibility varies markedly across isolates and antigen formats. These observations support the conclusion that the assay preserves native conformational constraints and reveal clear strain−specific differences in ADCP activity based on epitope accessibility. Because antigen presentation differs fundamentally across virion− and gp120−based formats, we did not perform quantitative cross−platform comparisons; instead, statistical analyses were conducted within each assay relative to matched negative controls. Although several antibodies showed significant ADCP activity, the magnitude and consistency varied, highlighting the importance of considering antigen context and biological effect size rather than relying solely on p−values.

Importantly, the virion−based assay performed robustly across diverse experimental conditions. It detected phagocytic activity mediated by broadly neutralizing and non−neutralizing monoclonal antibodies, polyclonal sera from people living with HIV, and vaccine−elicited antibodies in mice. The assay was reproducible across independent virus preparations, compatible with murine antibodies, and suitable for preclinical vaccine testing. However, there are several limitations to this study. The generation of virions and the use of ultracentrifugation introduce labor− and resource−intensive steps that may limit broader adoption of the assay. Nonetheless, the use of virion−derived Env remains essential for preserving native glycosylation, quaternary structure, and membrane−embedded features that recombinant Env proteins do not fully recapitulate. Alternative concentration approaches such as polyethylene glycol (PEG) precipitation, tangential flow filtration (TFF), or lower−speed cushion centrifugation may offer practical substitutes for ultracentrifugation, although each method would require empirical validation to ensure compatibility with this assay’s readouts and preservation of Env antigenicity. While THP−1 cells served as a standardized and scalable effector population, they do not capture the genetic diversity or phenotypic complexity of primary human monocytes and macrophages. Because phagocytic capacity and Fc receptor expression vary substantially across donors and myeloid subsets, future studies incorporating donor-derived primary myeloid cells will be important for defining the *in vivo* relevance and inter-individual variability of these findings. In addition, Env glycosylation is cell−type dependent and virions produced in 293T cells may not reflect the glycan heterogeneity found in primary CD4^+^ T cells or tissue−resident cells. As this study was designed as a proof−of−concept to establish assay feasibility, we used 293T−derived virions for consistency; however, future work will explore the use of virions derived from primary cells or alternative producer lines to assess glycosylation−dependent variability in ADCP readouts.

Together, our results establish the virion−based ADCP assay as a practical and biologically relevant tool for quantifying antibody−mediated phagocytosis of intact HIV−1 particles. By preserving critical features of native Env while enabling standardized, high−throughput evaluation, this approach complements existing ADCP methods and supports more nuanced analysis of Fc−mediated effector functions. Beyond HIV−1, this platform may be adaptable to other enveloped viruses for which native glycoprotein organization plays a central role in antibody function.

## Data Availability

The datasets presented in this study can be found in online repositories. The names of the repository/repositories and accession number(s) can be found in the article/**Supplementary Material**.
