# Supplementary figures and images for: Virus particle-based antibody-dependent cellular phagocytosis assay for HIV
Source: Front Immunol. 2026 Apr 24;17:1809342. doi: 10.3389/fimmu.2026.1809342 (PMC13153096; doi:10.3389/fimmu.2026.1809342)

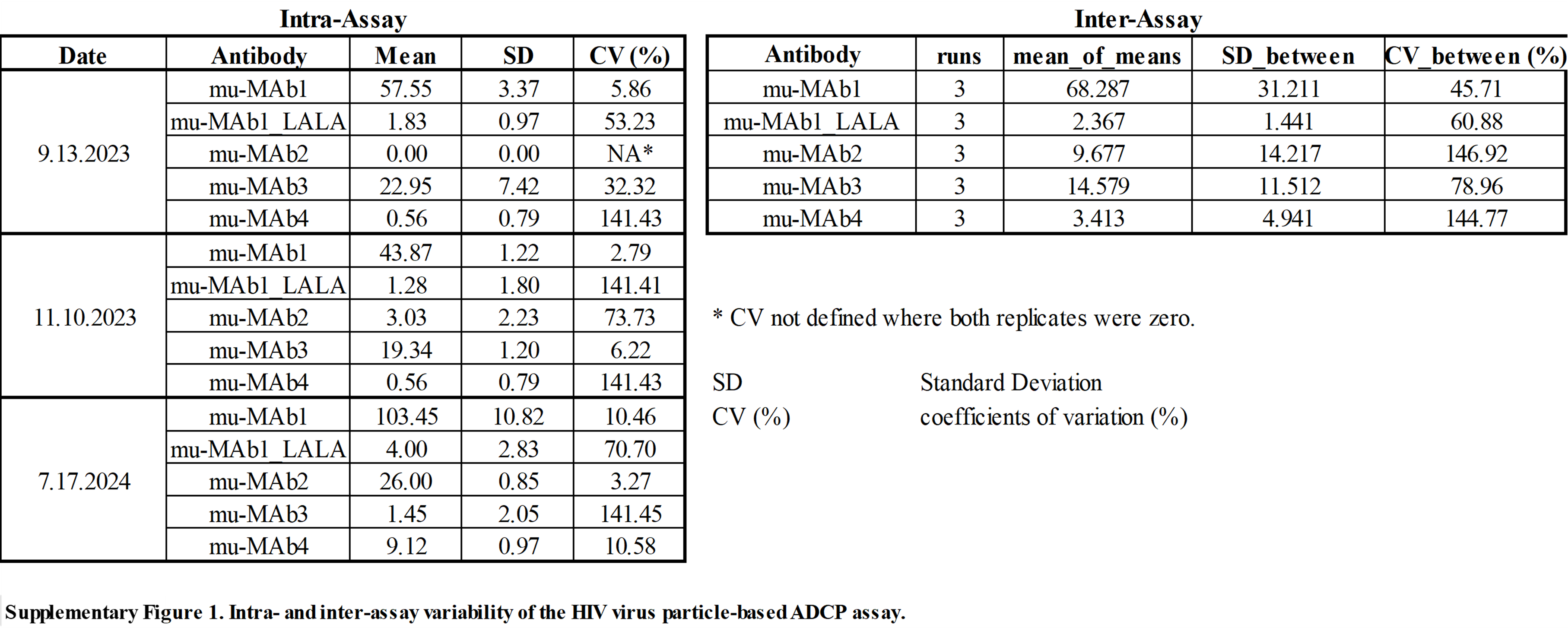

Supplement: Supplementary file 1 [file Image1.tif]
